# Humoral and Cell-Mediated Immune Response Validation in Calves after a Live Attenuated Vaccine of *Babesia bigemina*

**DOI:** 10.3390/pathogens9110936

**Published:** 2020-11-11

**Authors:** Umber Rauf, Muhammad Suleman, Asadullah Abid, Hamna Jamil, Harish Menghwar, Aneela Zameer Durrani, Muhammad Imran Rashid, Haroon Akbar

**Affiliations:** 1 Department of Parasitology, University of Veterinary and Animal Sciences, Lahore 54000, Pakistan; Umberrauf@gmail.com (U.R.); asadullah.abid@hotmail.com (A.A.); hamna1495@gmail.com (H.J.); drharoonakbar@uvas.edu.pk (H.A.); 2 Veterinary Research Institute (VRI), Lahore 54810, Pakistan; 3 University Diagnostic Laboratory, University of Veterinary and Animal Sciences, Lahore 54000, Pakistan; 4 Institute of Microbiology, University of Veterinary and Animal Sciences, Lahore 54000, Pakistan; 5 Vaccine and Infectious Disease Organization, International Vaccine Center (VIDO-InterVac), Saskatoon, SK S7N 5E3, Canada; ham776@usask.ca; 6 Molecular Immunology & Vaccine Development of Infectious Diseases, University of Saskatchewan, Saskatoon, SK S7N 5A2, Canada; 7 Department of Clinical Medicine and Surgery, University of Veterinary and Animal Sciences, Lahore 54000, Pakistan; aneela@uvas.edu.pk

**Keywords:** *Babesia bigemina*, attenuated vaccine, humoral immunity, cell-medicated immunity, CD4+ T cells, CD8+ T cells, bovine babesiosis

## Abstract

The current vaccines to control bovine *Babesia bigemina* (*B. bigemina*) infection are not fully protective and vaccination failures incur heavy losses to the cattle industry around the world. Using modified micro-aerophilous stationary phase, we developed a culture-derived attenuated live vaccine against *B. bigemina* and tested a single subcutaneous inoculation of 2 × 10^8^ infected erythrocytes in calves. The protection was measured after a lethal intravenous challenge with 5 × 10^8^ virulent calf-derived *B. bigemina*. Our results demonstrated that a single shot of attenuated vaccine was capable of inducing robust humoral and cell-mediated immune responses in calves. We found a significant increase in the IgG antibody titers post-challenge and a strong proliferation of both CD4+ and CD8+ T cells contributing towards the protection. Our vaccine provided complete protection and parasitic clearance, which was followed for more than 100 days post-challenge. This immunity against babesiosis was directly linked to strong humoral responses; however, the parasitic clearance was attributed to significant T cells effector responses in vaccinated calves as compared to the infected control calves. We anticipate that these results will be helpful in the development of more efficient culture-derived vaccines against *Babesia* infections, thus reducing significant global economic losses to farmers and the cattle industry.

## 1. Introduction

Bovine babesiosis is an emerging disease that proves not only fatal but also causes enormous economic loss worldwide in many ways—such as direct treatment cost, the indirect cost of low meat production and loss of milk production [1]. Globally, there are around 1.2 billion cattle that are at risk of being infected with babesiosis including in Asia, Australia, USA, Africa, Central and South America [2]. According to the Food and Agriculture Organization (FAO), 80% of the world’s cattle population is exposed to tick infestation and it has estimated an impact of 7.3 US $/head/year [3]. *Rhipicephalus microplus* is a significant tick for the cattle industry all over the globe, causing almost USD 22–30 billion in losses per year [4] by the transmission of serious pathogens in animals [5].

It is well documented that widely used chemotherapeutic compounds like diminazene aceturate and imidocarb dipropionate have an ample spectrum against this apicomplexan hemoprotozoan pathogen for bovine babesiosis [6,7]; but the repeated use of these drugs has led to the development of drug resistance in the tick-borne hemoprotozoan parasite [8,9], which has residual impact on milk and meat for a longer period, even after treatment [10]. The aforementioned drugs are banned or restricted by the Food and Drug Administration in many countries [11]. This drug resistance development highlights the need for the exploration of new pharmaceutical agents with effective babesicidal activity and low toxicity in the host [12,13].

It is well established that in future protection against babesiosis can be achieved by better vaccination of cattle in order to control the disease before it occurs [14]. Vaccines that are presently employed in Australia, South Africa, several countries of South America and Israel [15,16,17,18,19,20] consist of bovine blood containing attenuated parasites. However, by no means all of these attenuated parasites are completely avirulent. It is generally known that, because of the wide diversity of *Babesia* spp. [21] in different geographical regions, it would be preferable to use autochthonous strains to obtain a highly potent vaccine. Besides, the introduction of new exotic strains, or new genetic/antigenic variants [22] into a geographical area, might present a serious risk for the severity of the disease status in the field. The calf derived vaccines developed by ‘‘slow’’ passages in vivo in intact calves [23] were shown to be highly effective but are known to have the potential of transmitting latent pathogens if testing is insufficiently stringent [24].

Following the introduction of in vitro culture technology for *Babesia bigemina* [25], culture-derived vaccines, which present minimal risk of the inadvertent spread of pathogens, were adopted and showed immunogenicity comparable with that of calf-derived vaccines [14,17,18,26]. In countries with no possibility of maintaining cattle under highly stringent conditions, vaccines derived from an in vitro culture are a suitable alternative source for parasites used for immunization [27]. Recently, Tick Fever Center, Wacol, Australia has commercialized Tick fever vaccine against *B. bigemina* and *B. bovis*, with a short shelf life and suitable for the local environment [28]. Frozen culture derived parasitic vaccines have shown promising results in various field trials in Mexico [29], whereas others have tried co-immunization of live attenuated *B. bovis* and *B. bigemina* vaccines with *Lactobacillus casei* to improve the parasite specific IgG1 levels [30]. A comprehensive review published on in vitro cultivation of *Babesia* aimed exclusively at the purpose of live attenuated vaccine production demonstrated the importance of the advancement of culture-derived attenuated vaccines to control bovine babesiosis [31].

## 2. Materials and Methods

### 2.1. Ethical Statement

The current study was approved by the animal welfare and ethics society of the University of Veterinary and Animal Science, Lahore, Pakistan with No. DR 1112, Dated: 13 October 2017.

### 2.2. Source of Infection

The local strain of *Babesia bigemina* was isolated form an infected calf located on a farm near the city of Lahore (31.4340° N, 74.1945° S). The infected erythrocytes were visualized on thin smear and validated by species-specific PCR methods. We also reared *B. bigemina*-infected colonies of *Rhipicephalus microplus* at 28 °C temperature and at humidity >80% in a Biological Oxygen Demand (BOD) incubator (Model ICO105 Memmert, Germany) which were fed periodically on cattle host. Two six-month-old calves (non-splenectomized) were infested with *B. bigemina*-infected *Rhipicephalus microplus*. Thin blood smears of their samples were prepared to examine the Percentage Parasitized Erythrocytes (PPE) of the infected calves. To calculate the parasitemia level in infected calves and in cultured red blood cells (RBCs), three smears from each sample were prepared to count the average number of *B. bigemina*-infected RBCs. Parasitemia level was calculated according to the previously reported formula [32]. After experimentation, the morbid animals were medicated. (Parasitemia = log (infected RBCs/10^5^ erythrocytes)).

### 2.3. Genomic DNA Extraction and PCR Assay

A pair of oligonucleotides was selected for the identification of *B. bigemina* as described earlier [33]. Genomic DNA was extracted from 200 µL of blood samples through a DNA extraction kit (GeneAll^®^, Exgene™, 105-101) according to the manufacturer’s instructions, while a quantity of DNA was measured through NanoDrop spectrophotometer (Thermo Scientific 2000/2001, Wilmington, DE USA). PCR was used for confirmation of *B. bigemina* by using specific primer pairs: Bg-forward: 5′-GTATCAGCCGCCGACCTCCGTAAGT-3′ and Bg-reverse: 5′- GGCGTCAGACTCCAACGGGGAACCG-3′. PCR was performed according to the protocol described previously [33] with slight modifications. Briefly, PCR reaction was carried out in 20 µL of reaction mixture containing 1 µL of each primer pair (10 pmol), 2 µL of DNA, 10 µL of 2× AmpMaster™ Taq (GeneAll^®^, Exgene™, 541-001) and 6 µL of UltraPure™ DEPC water (Cat no. 750023; Invitrogen, Carlsbad, CA, USA). The positive control DNA extracted from known *B. bigemina* positive sample was run with each reaction. The PCR cycle was initiated by initial denaturation at 95 °C for 5 min, followed by 35 cycles, comprising of 95 °C for 30 s, annealing at 68 °C for 1 min and extension at 72 °C for 1 min. The final elongation step was performed at 72 °C for 10 min, followed by agarose gel electrophoresis in 1.5% (120 V, 200 mA, 45 min) stained with ethidium bromide (Cat no. 15585-011; Invitrogen, Carlsbad, CA, USA) and observed under a GelDoc 100 imaging system. DNA ladder of 100 bp (Genedirex, Catalogue # DM001-R500) was used to compare the amplified product of 738 bp specific for *B. bigemina*.

### 2.4. Attenuation of Local Strain and Vaccine Preparation

*B. bigemina* parasites were subjected to in vitro passages for attenuation through the modified micro-aerophilous stationary phase (MASP) technique [34]. On the confirmation of infected calves, blood samples (10 mL each calf) were collected for in vitro propagation of the parasite as described elsewhere [35]. Briefly, the subcultures were performed in 24-well culture plates (Corning, New York, NY, USA) using Medium 199 (Thermo Fisher, Helsinki, Finland) supplemented with fetal bovine serum (40%), penicillin (100 IU), streptomycin (100 μg/mL), HEPES (15 mM) and amphotericin B (50 μg/mL). The cultures were incubated at 37 °C with 5% CO_2_ and after every 72 h, the cells were washed three times using PBS (0.1 M) and re-suspended in fresh culture medium. After about 20 in vitro passages, with the cultured parasites at 10% parasitemia in the Giemsa-stained smears, their pathogenicity was determined by inoculating them into naïve calves. The calves were evaluated on the basis of characteristic clinical signs of acute babesiosis (pyrexia, hemoglobinuria, anemia and packed cell volume decrease, Jaundice). No clinical signs were observed in the calves inoculated with attenuated parasites. A culture-derived vaccine was prepared by propagating the attenuated parasites in 225 cm^2^ Corning^®^ cell culture flasks (Merck, New York, NY, USA). The vaccine doses contained 4 × 10^8^ culture-derived infected erythrocytes frozen in a total volume of 2 mL having 15% DMSO (Dimethyl sulfoxide) in liquid nitrogen. The vaccine doses thawed at 40 °C and 2 mL PBS having 15% DMSO was added before inoculation.

### 2.5. Animal Trial

Twelve calves (6–8 months old) were used for vaccination and challenge trials. The calves were placed in research pens at University of Veterinary and Animal Sciences, Lahore (UVAS) and acclimatized for 10 days, received “ad libidum” food and water and tested for *B. bigemina* negative by blood smear and PCR screening. The calves were randomly divided into three groups. Group A (three calves) was kept as a healthy control group for measuring the baseline antibody titers, CD8+ and CD4+ T cell populations. Group B (six calves) was inoculated subcutaneously with 2 mL of vaccine dose containing 2 × 10^8^ culture-derived infected RBCs (containing attenuated parasites) and served as a vaccinated group. Group C (three calves) was the infected control and served as an acutely challenged group without any vaccination. The vaccination and challenge were performed according to the protocol described previously, with modifications [35]. Briefly, the animals of vaccinated group B were challenged intravenously two months after the immunization, with 5 × 10^8^ calf-derived virulent *B. bigemina* infected RBCs. The group C infected control was challenged simultaneously with the group B calves. The vaccinated and infected animals were monitored daily by animal care staff for signs of depression, drop in feed intake and rise in rectal temperature. The survival of different groups was recorded up to day 100 post-challenge. All calves were reared with free access to water and corn silage during the trial.

### 2.6. Preparation of Native Antigens

Merozoites of *B. bigemina* were harvested from iRBCs according to the protocol described elsewhere [36] with modifications as follows. Twenty milliliters of whole blood was centrifuged for plasma and erythrocyte separation at 250× *g* (BIOShield Swing-out Bucket Rotor, Catalogue # 75003182, ThermoFischer^®^) at 4 °C for 3 min. Supernatant was discarded and the pellet of cells was washed thrice with 1× Phosphate Buffered Saline (PBS) following centrifugation at 1000× *g*. Immediately after centrifugation, the pellet was treated with three parts of cold ammonium chloride lysis buffer (0.17 M) for one minute (Podoba and Stevenson, 1991). Reaction was stopped by adding RPMI-1640. The mixture was centrifuged at 1000× *g* for 15 min and the erythrocyte-free pellet was washed three times in PBS. The pellet was resuspended in 5 volumes of PBS containing protease inhibitor (1 mM PMSF, 2 mM TPCK and 0.1 mM TLCK). *B. bigemina* merozoites were disrupted by repeated freeze/thaw method in liquid nitrogen. The supernatant obtained after centrifugation at 10,000× *g* for 1 h at 4 °C was stored at −20 °C. Quantification of the Ag was assessed through BCA Kit (Bicinchoninic Acid) (Cat. 786-570, G-Biosciences^®^), following the manufacturer’s protocol.

### 2.7. Serology

ELISA was performed to detect bovine immunoglobulin (IgG) according to the protocol described elsewhere [36,37]. Briefly, 10 µg/mL of native antigens were coated in a 96 well ELISA plate (BIOFIL^®^, Guangzhou, China) in 50 mM bicarbonate buffer, incubated at 4 °C overnight. The ELISA plates were washed three times with washing buffer (0.05% Tween 20, 0.01 M PBS, PH 7.2). Saturation of the microtiter plate was performed with 4% BSA in PBS followed by incubation at 37 °C for 2 h. Both negative and positive sera were diluted in PBS to achieve two-fold serial dilutions. Diluted sera were poured into each well. The plate was incubated again at 37 °C for 1 h. The second washing was performed with a washing buffer as described above. Bound antibodies were detected by incubating at 37 °C for 2 h with goat anti-bovine IgG-alkaline phosphatase conjugate (1:10,000). After washing thrice, phosphatase activity was measured with P-nitrophenyl phosphate (pNPP, Cat. 41480004-1, Bioworld^®^, Dublin, OH, USA) at 1 mg/mL in 1 M diethanolamine (Cat. 40400060-3, Bioworld^®^). Optical density (OD) values were obtained by ELISA reader (Elisa reader, Model ELx 800, BioT, Winooski, VT, USA) at a wavelength of 405 nm.

### 2.8. Blood Cell Isolation for Flow Cytometry

Whole blood was collected from the right jugular vein of calves from groups A, B and C. The procedure for blood processing for flow cytometry (FCM) was performed as described earlier [38]. Briefly, a total of 10 mL of blood was collected in syringes with EDTA (1.8 mg per mL blood). Blood was transferred to falcon tubes and centrifuged at 2400× *g* for 20 min at 4 °C with no brakes. The top layer of plasma was discarded and the buffy coat layer was collected. The buffy coat cells were mixed with PBS and then re-centrifuged at 2400× *g* for 20 min with no brakes. We removed and discarded the supernatant and lysis of red blood cells was performed. The cells were incubated with a lysis solution for 10 min and re-centrifuged as mentioned above. The pellet obtained after centrifugation was suspended in PBS. The cells were counted, and cell viability was over 95% as determined by trypan-blue exclusion method.

### 2.9. Antibodies

Mouse-anti-bovine monoclonal anti-CD4 antibody (Cat # MA1-80176; Thermo Fisher Scientific, USA) was used for flow cytometric detection of bovine CD4+ T cells (1:100 dilution) and mouse-anti-bovine monoclonal anti-CD8 antibody (Cat # MA1-80900; Thermo Fisher Scientific, USA) was used for flow cytometric detection of bovine CD8+ T cells (1:100 dilution). A non-specific mouse IgG2a (Cat # PA5-33239; Thermo Fisher Scientific, Waltham, MA, USA), used at the same concentration as the primary antibodies, was employed as an isotype control.

### 2.10. Flow Cytometry

Flow cytometry was used to quantify bovine CD4+ and CD8+ T cells. The counted cells aliquot of 5 x 10^5^ cells per tube for each sample and animal were blocked by incubation on ice for 30 min in PBS + 4% BSA. The final staining with antibodies was done in 100 μL PBS + 1% BSA. Every monoclonal antibody concentration was titrated to obtain the best staining intensity without background staining. The cells were incubated in the dark with the monoclonal antibody on ice for 30 min and later washed twice by adding 500 μL PBS + 1% BSA. Cells were then fixed with 200 μL of 2% paraformaldehyde (SigmaAldrich^®^ St. Louis, MO, USA), before proceeding with flow cytometry. A FACS AttuneX^TM^ NxT acoustic focusing cytometer (Invitrogen^®^, Waltham, MA, USA) was used for sample acquisition and 10,000 events were acquired per sample and analyzed with NxT software (version 2.7).

### 2.11. Statistical Analyses

The antibody titers and FCM quantification of CD8+ and CD4+ T cells were assessed with Mann-Whitney test when comparing two groups and Kruskal–Wallis test when comparing more than two groups. Statistical differences were considered significant at *p* ≤ 0.05 a priori. Data were analyzed using GraphPad Prism 7 for Mac OS X (GraphPad Software, La Jolla, CA, USA, www.graphpad.com).

## 3. Results

### 3.1. Microscopic and PCR Detection

**Figure 1:**
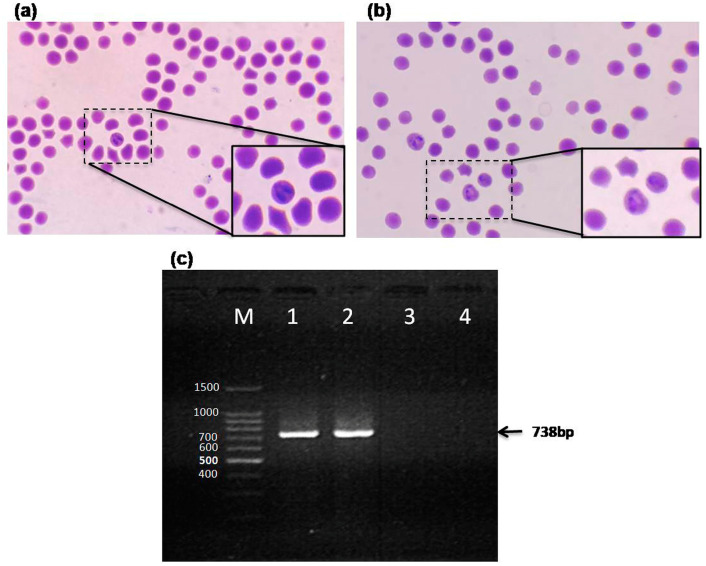
*Babesia bigemina* detection using bright field microscopy and PCR. (**a**) Microscopic examination at 1000× magnification (100× oil emersion lens with 10× eye piece) showing Babesia-like (pear shaped piroplasm) in the erythrocytes (dotted box), with an inset box showing close up of infected cell from group C. (**b**) Blood smear from vaccine trial group B calves (post-challenge) showing multiple *Babesia*-infected erythrocytes (dotted box) and inset box showing close up of infected cells. (**c**) Agrose gel showing PCR product size 738 bp amplicons specific for *B. bigemina* (black arrow). Lanes 1 and 3 show control positive *B. bigemina* DNA and control negative DNA extracted from uninfected blood sample, while lane 2 and 4 show representative vaccine trial samples from Group C and Group A. Lane M shows 100 bp DNA ladder.

Microscopic blood smear examination and PCR was performed for animal screening before starting the animal trial. The infected control calves from group C showed intra-erythrocytic pear shaped piroplasmic bodies (Figure 1a). The representative image from group B calves showing multiple *Babesia*-infected erythrocytes (Figure 1b). The parasitemia levels observed in *B. bigemina* infected control calves in group C were at 3% ±0.5, whereas the parasitemia levels in vaccinated calves post-challenge were at 0.8% ±0.2. The parasitemia in Group B was observed till day 12 post-challenge and afterwards a clearance was recorded up to day 100 post-challenge. Confirmation of *B. bigemina* was accomplished through a highly specific PCR at different time points of the animal trial. The PCR product size of 738 bp was obtained as shown in Figure 1c.

### 3.2. Flow Cytometric Quantification of CD4+ and CD8+ T Cells in Control Calves (Group-A)

**Figure 2:**
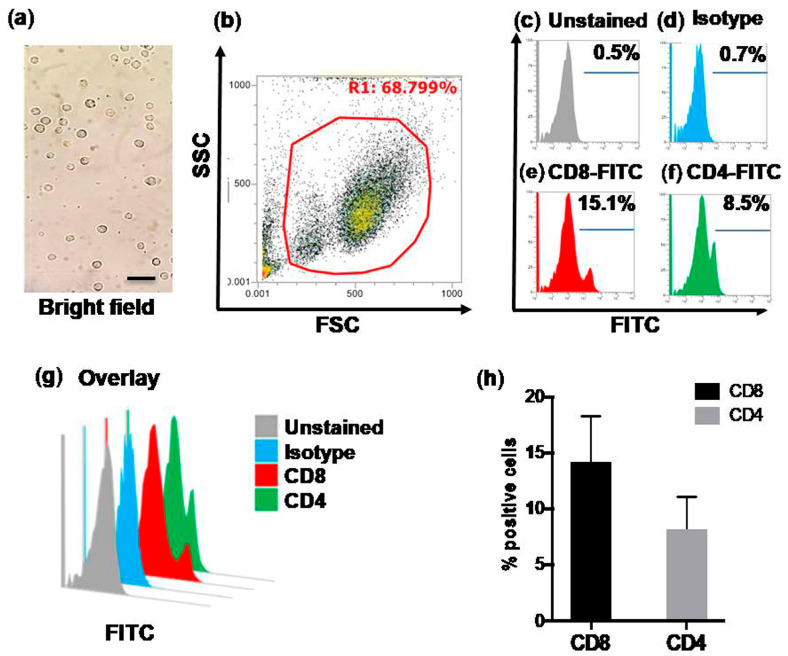
Detection and quantification of bovine CD4+ and CD8+ T cells in Group A healthy uninfected calves using flow cytometry (FCM). (**a**) Bovine blood was processed and buffy coat cells were separated and counted. The cells were blocked using 4% BSA for 30 min and the staining of cells using a specific antibody. (**b**) Attune™ NxT Acoustic Focusing Cytometer was used for flow cytometry. Scatter plot showing gating of total Bovine buffy coat cells, (**c**) Histogram showing unstained cells, (**d**) Histogram showing cells stained with isotype control, (**e**) Histogram showing CD8+ FITC stained cells, (**f**) Histogram showing CD4+ T-FITC stained cells, (**g**) Overlay plot showing unstained, isotype control, CD8+ and CD4+ positive populations. (**h**) Bar graph showing data from three control uninfected calves. Data is representative of three independent experiments with each condition performed in triplicate. FSC = Forward scatter, SSC = Side scatter, FITC = Fluorescein Isothiocyionate. Scale bar = 100 μm.

To evaluate the quantities of CD4+ and CD8+ T cells in healthy control calves in group A, we isolated buffy coat cells from whole blood and enumerated them under a bright-field microscope before staining for FCM (Figure 2a). The purified bovine buffy coat cells were then processed through Attune NxT and adjustment of forward and side scatter of cells was performed for gating (Figure 2b). The fluorochrome intensity (in BL-1 filter) was adjusted using unstained cells (Figure 2c) and isotype control labeled cells (Figure 2d). We found 15.1% of CD8+ T cells (Figure 2e) and 8.5% of CD4+ T cells (Figure 2f). The overlays were generated using NxT software to show unstained, CD8+ and CD4+ T cells (Figure 2g). The bar graph was plotted for percentage positive CD8+ and CD4+ T cells found in healthy calves (Figure 2h).

### 3.3. Subcutaneous Immunization with Culture-Derived Attenuated B. bigemina Elicited Increased CD4+ and CD8+ T Cells Responses

**Figure 3:**
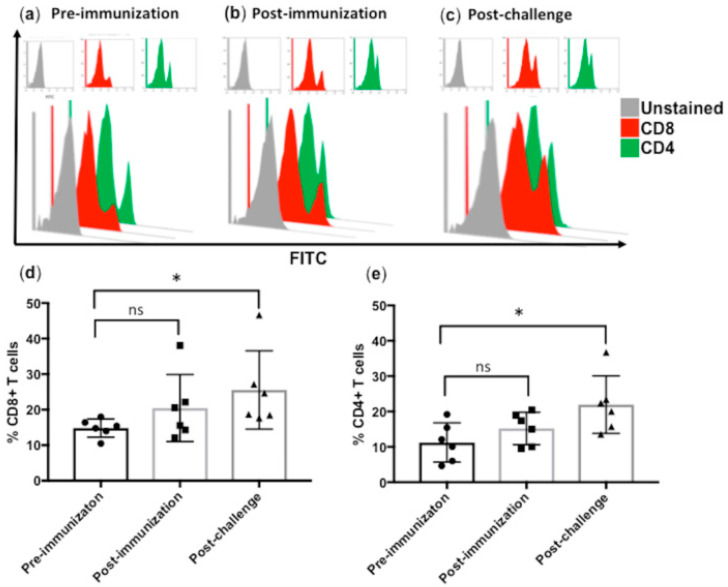
CD4+ and CD8+ T cells quantification in Group B vaccinated calves during pre-immunization, post-immunization and post-challenge stage. (**a**) Histograms of unstained (Grey), CD8+ (Red) and CD4+ (Green) stained T cells and their overlay for calves before vaccination (pre-immunization), (**b**) Histograms of T-cells and their overlay after vaccination (post-immunization), (**c**) Histograms of T-cells and their overlay after challenge of vaccinated calves (post-challenge), (**d**) Bar graph showing a significant increase in the CD8+ T cells in post-challenge, (**e**) Bar graph showing a significant increase in the CD4+ T cells in post-challenge. Bar graph showing representative data from three independent experiments. Each individual animal had each treatment performed in triplicate, and the bar graphs show representative data as medians with error bars. Significant results are shown at *, *p* < 0.05, ns = not significant.

The single dose of subcutaneous immunization leads to an efficient increase in the T-cell responses in Group B vaccinated calves. We conducted FCM for quantification of CD4+ and CD8+ T cells at pre-immunization (Figure 3a), 7 days post-immunization (Figure 3b) and 21 days post-challenge (Figure 3c). We evaluated the calves at the pre-immunization stage to set-up a base-line quantification before vaccination, and we found a trend towards an increase in the number of both CD4+ and CD8+ T cells post-immunization; however this increase was found to be statistically significant (Figure 3d,e). We then quantified the CD4+ and CD8+ T cells in calves post-challenge and found a significant increase in the number of T cells at the post-challenge stage of the vaccine trial when compared with pre-immunization counts (*p* < 0.05) (Figure 3d,e).

### 3.4. Significant Reduction in CD4+ and CD8+ T Cells in B. bigemina Challenged Calves of Group C

**Figure 4:**
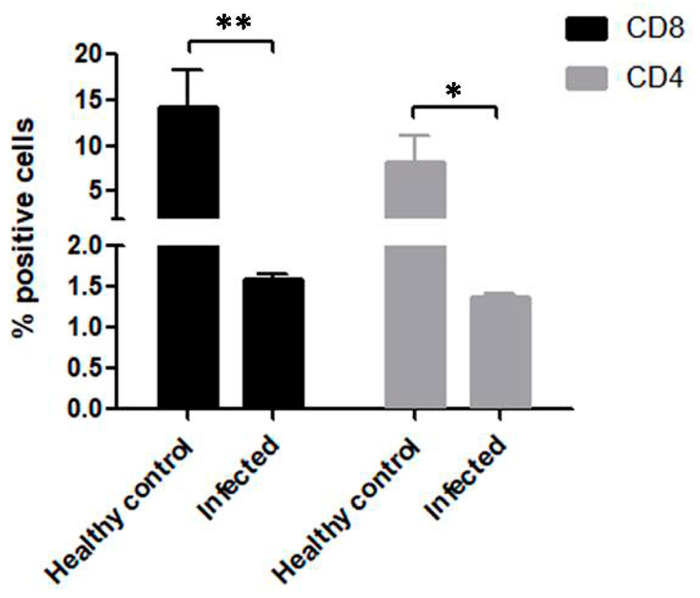
*Babesia bigemina* challenge induced a severe reduction in both CD4+ and CD8+ T cell quantities in non-vaccinated challenged Group C calves. The flow cytometry based quantification was performed and a significant reduction was observed as compared to healthy control Group A calves. Bar graph showing representative data from three independent experiments with conditions performed in triplicate showing medians with error. Significant results are shown at *, *p* < 0.05 and **, *p* < 0.01.

We quantified, using flow cytometry, the CD4+ and CD8+ T cells in blood collected from the Group C calves that were unvaccinated and challenged by virulent *B. bigemina* and compared them to healthy control calves in Group A. The FCM analysis showed CD8+ T cells decreased to 1.6% in the infected samples and the CD4+ T cells were found at 1.37% (Figure 4). We compared the cytometry data of the two groups to show the comparative difference and found significant difference in both CD4+ T cells (*p* < 0.05) and CD8+ T cells (*p* < 0.01) (Figure 4).

### 3.5. Comparison of CD4+ and CD8+ T Cells between Calves from Group A, B and C

**Figure 5:**
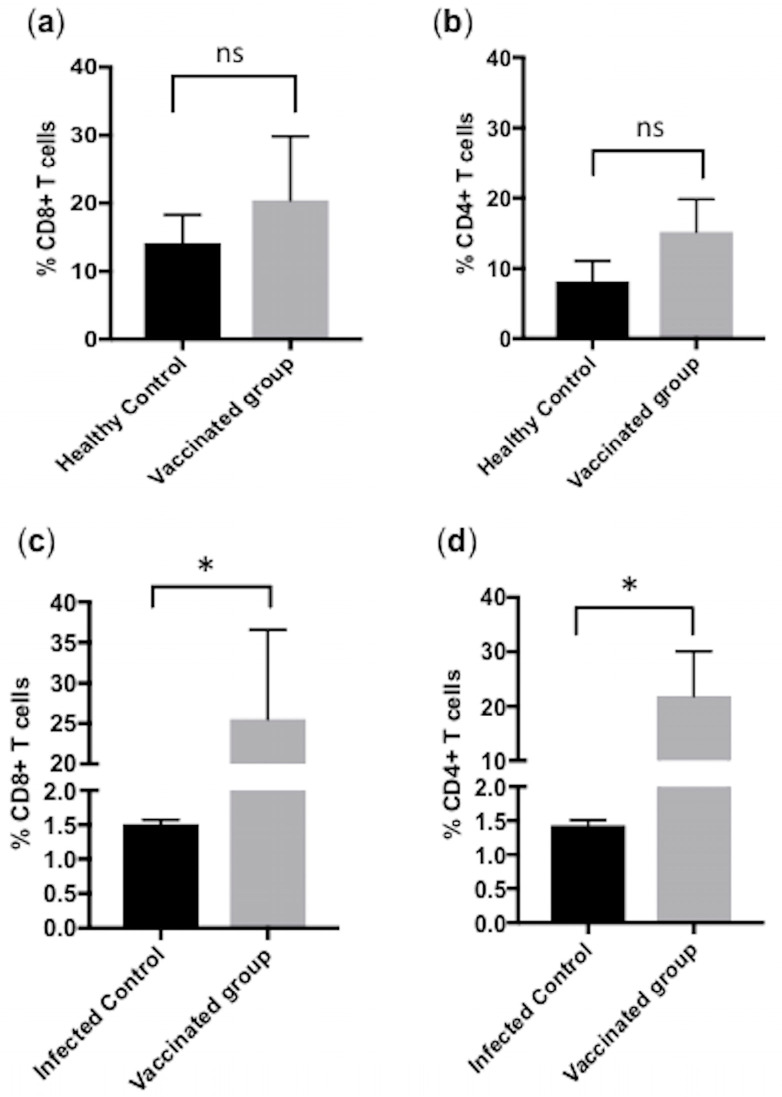
Comparison of CD4+ and CD8+ T cells in healthy control (Group A), infected control (Group C) and vaccinated calves (Group B). (**a**) The FCM quantification of CD8+ T cells of Group A healthy control calves and Group B vaccinated pre-challenge calves. (**b**) Bar graph of CD4+ T cells of Group A healthy control calves and Group B vaccinated pre-challenge calves. No significant increase was seen in both cell populations. (**c**) The Bar graph shows a statistically significant increase of CD8+ T cells in vaccinated group B post-challenge when compared with Group C challenged infected control calves without vaccination. (**d**) Bar graph comparing vaccinated group B post-challenge with Group C challenged infected control calves without vaccination showed a significant increase of CD4+ T cells in vaccinated calves. Data shown is representative of three independent experiments. Each individual animal had each treatment performed in triplicate, and the bar graphs show representative data as median with error. Significant results are shown at *, *p* < 0.05. ns = not significant.

In the vaccination trial, we addressed the establishment of cell-mediated immunity by comparing the CD4+ and CD8+ T cell quantities among the different groups. Our results demonstrated that there was a trend towards an increase in the amount of both CD4+ and CD8+ T cells between Group A calves (healthy control) and Group B calves (vaccinated pre-challenge); however, this increase was not statistically significant (Figure 5a,b). Concerning the comparison of Group B calves (vaccinated post-challenge) with the Group C calves (unvaccinated and challenged), we found a significant increase (*p* < 0.05) in the quantities of both CD4+ and CD8+ T cells in vaccinated post-challenge ones (Figure 5c,d).

### 3.6. Subcutaneous Immunization with Culture-Derived Attenuated B. bigemina Elicited Increased Humoral Response

**Figure 6:**
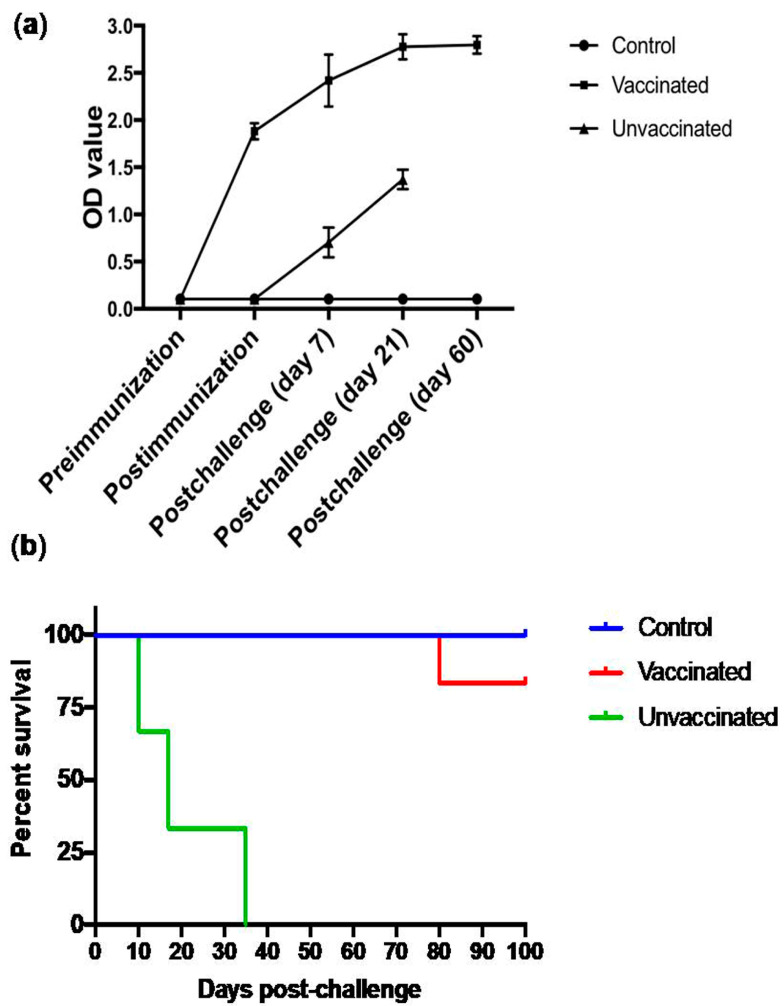
Induction of significant humoral immune response by different groups in vaccine trials and their survival post-challenge. (**a**) Line graph showing humoral response between Group A (Control), B (Vaccinated) and C (unvaccinated). Significant increase was found at day 21 and day 60 post-challenge in comparison to control Group A and Group B. (**b**) Bar graph showing percentage survival among different groups with no death in control group A, one calf lost in vaccinated group B and one death and two clinically critical, hence humanely euthanized. Representative data from three independent experiments. Each individual condition performed in triplicate. OD = Optical density (405 nm).

We evaluated the humoral response at different time points starting at Day 0 pre-immunization, Day 7 post-immunization, Day 7 post-challenge, Day 21 and Day 60 post-challenge. We found a steady increase over time and a significant difference between Group A vs Group B (*p* < 0.05). We also found that, within the vaccinated group, there is a significant difference between pre-immunization and Day 21 and Day 60 post-challenge (*p* < 0.05) (Figure 6a).

### 3.7. Animal Trial Outcome and Survival Curve

After the challenge, we observed the calves for clinical signs such as high fever, depression, anorexia and hematuria. The three calves from Group A remained healthy with no clinical signs or mortality. The six calves from vaccinated Group B were challenged and monitored, and no significant clinical signs were observed up to three months post-challenge. However, one calf from Group B died with non-Babesia-like clinical signs and a necropsy report showed death linked to enteric lesions. The three non-vaccinated challenged calves in Group C displayed severe clinical signs characteristic of acute Babesiosis and one calf died on day 10 post-challenge, whereas the other two calves were humanely euthanized due to adverse clinical signs on day 18 and day 35 post-challenge (Figure 6b).

## 4. Discussion

Bovine babesiosis is considered as a top arthropod-transmitted disease of bovines, and researchers around the globe are struggling to reduce the economic burden linked to this disease [39]. Temporal and spatial analysis revealed increased incidence over the years and the possibility of spread to new areas of the world [40]. Currently, babesiosis is prevalent across six continents infecting 62 countries and causing major losses to the livestock sector [40]. The highest prevalence was reported in South America (64%) and *Babesia bigemina* was found to be the highest prevalent among all other cattle diseases [40].

The disease is being partly controlled using therapeutic drugs or local vaccines but with limited success; hence more in-depth research is needed to develop a better understanding of its pathogenesis in order to eradicate this disease from bovine herds. It has been established that there exists a gap in knowledge of how *Babesia* parasite interacts with immune cell populations and how the parasite can generate acute infection with clinical signs, and also generate persistent infection status without evident clinical symptoms, helping in long-term survival in the host [41].

We know that many bovine pathogens including viruses, bacteria and parasites inhibit immune proliferation of CD4+ and CD8+ T cells leading to the development of poor cell-mediated immunity. Bovine leukemia virus causes progressive exhaustion of T cell functions by up-regulation of programmed death-1 (PD-1) and lymphocyte activation gene-3 [42], and blockage of its receptor PD-L1 leads to enhancement of antiviral responses [43]. Certain bovine respiratory complex pathogens like *Mycoplasma bovis* strongly inhibit lymphoproliferation of CD4+ and CD8+ T cells during infection [44]. *Anaplasma marginale* is also known to downregulate antigen-specific T and B cells leading to a failure to establish effective memory T cell responses and persistent infection [45]. There have been several studies which indicate that strong T lymphocyte activation is needed to confer protection against *Babesial* infections [46,47,48,49], malarial infections [50] and *Theilerial* infections [51].

In accordance with the above-mentioned findings, we set out to investigate the quantities of CD4+ and CD8+ bovine T cells during the course of *Babesia* infection and enumerate them during our new culture-derived attenuated live vaccine trial as a hallmark of protection [52]. We found an acute depletion of both CD4+ and CD8+ T cell populations in *B. bigemina* challenged calves (Group C) as compared to healthy calves (Group A) and their up-regulation in the vaccinated calves (Group B). This phenomenon of parasite-induced T cell depletion can directly be correlated to acute infection and poor elimination of infected cells from circulation [53]. This has also been observed in other parasites like *Trypanosoma cruzi*, *Leishmania sp.*, *Toxoplasma gondii* and *Plasmodium sp.* where CD8+ T cell mediated cytolysis of infected cells is considered critical for long-term resistance and effective vaccine development [54,55]. The depletion of CD4+ T cells in acute infection was also indicative of poor protection against the parasite, as a similar phenomenon was reported for *Toxoplasma gondii* infection where CD8+ T cell dysfunction was linked to CD4+ T cell exhaustion [56].

Our vaccine with single immunization not only induced long-lasting humoral response but also strongly induced both CD4+ and CD8+ T cell proliferation contributing synergistically towards parasite clearance, whereas other vaccine studies using viral vectors required a prime boost strategy with multiple vaccine shots to induce substantial immune responses [57]. The induction of immune responses to live-attenuated vaccines is known to buildup gradually as compared to the recombinant vaccines [58]. This was observed for our vaccine trials where both CD4+ and CD8+ T cells showed a gradual increase from pre-immunization to post-immunization status. The FCM quantification performed at day 7 post-immunization could have been too early to record a significant increase in cellular responses; however, a promising boost in both humoral and cellular responses was observed post-challenge. A similar effect was observed in an increasing trend towards differences in CD4+ and CD8+ T cells between healthy control calves (Group A) and vaccinated calves (Group B), but most importantly there was a significant difference between infected control calves (Group C) and vaccinated calves (Group B) elucidating their potential role in protection.

It has already been established over the years that control of parasitemia in bovine babesiosis is dependent on clearance of *Babesia*-infected erythrocytes by splenic macrophages, which are activated by a fine balance between CD4+ and CD8+ T cells in circulation [59]. It is necessary that higher CD4+ T cells availability will be able to provide significantly higher assistance to CD8+ T cells and other immune cells in performing adequately enough to reduce intra-cellular parasite burden in order to block disease systems and completely clear the infection [60]. Hence, the significant higher induction of both CD4+ and CD8+ T cells in our study at the post-challenge stage is directly associated with the absence of parasitemia on blood smear and PCR in Group B vaccinated calves up to day 100 post-challenge. We also observed a significant boost in antibody titers post-challenge that are well known to directly neutralize extracellular merozoites [59]. The mechanism(s) involved in the depletion of CD4+ and CD8+ T cells during *Babesiosis* are likely to be linked with molecular signaling pathways induced by the *Babesia* parasite orchestrating the infection. The present study was not designed to explore molecular interactions but envisions a significant interest for future studies to find molecular targets for interventional therapy in controlling this parasitic infection in bovines.

Collectively, our findings provide novel evidence of the role of both bovine CD4+ and CD8+ T cell populations in bovine *Babesiosis,* as their depletion, seen during the challenge, leads to the development of acute babesiosis. To our knowledge, this is the first study of flow cytometric quantifications of bovine CD4+ and CD8+ T cell during *B. bigemina* vaccine trials as many previous studies focused on functional assays rather than critical absolute quantifications of immune cells induced post-vaccination and post-challenge. Our culture-based attenuated live vaccine from local *B. bigemina* isolate provided complete protection of vaccinated calves and has the potential for playing a vital role in controlling this infection in bovine herds and reducing economic losses for farmers linked to the dairy and meat industry.
